# Barriers and facilitators to older adult participation in intergenerational physical activity program: a systematic review

**DOI:** 10.1007/s40520-023-02652-z

**Published:** 2024-02-12

**Authors:** Fan Zhou, Hong Zhang, Hong Yan Wang, Lin Feng Liu, Xian Geng Zhang

**Affiliations:** 1 Chengdu University of Traditional Chinese Medicine, Chengdu, 610075 Sichuan China; 2 Sichuan Nursing Vocational College, Chengdu, 610100 Sichuan China

**Keywords:** Intergenerational interactions, Physical activity, Older adults, Barriers, Facilitators, Systematic review

## Abstract

**Background:**

The intergenerational physical activity program aims to promote the health, social engagement, and well-being of older adults. It is essential to comprehend the barriers and facilitators that affect their involvement to develop successful intervention strategies. This systematic review critically examines available research to identify the factors that impact the participation of older adults in intergenerational physical activity programs.

**Methods:**

This study retrieved 13 electronic databases (from January 2000 to March 2023) and used a social-ecological model to classify and analyze the identified facilitators and barriers.

**Results:**

A total of 12 articles were included, which identified 73 facilitators and 37 barriers. These factors were condensed into 7 primary themes and 14 sub-themes in total.

**Conclusions:**

The factors influencing the participation of older adults in intergenerational physical activities are multifaceted. These factors guide project developers, policymakers, and practitioners in developing and implementing intergenerational physical activity programs to help address global aging issues and promote intergenerational connections.

**Trial registry:**

PROSPERO ID: CRD42023420758.

## Introduction

In the context of global aging, promoting physical activity (PA) [1] among older adults has become an urgent public health priority. The World Health Organization has developed specific guidelines for physical activity in older adults [1], recommending that older adults engage in at least 150 min of moderate to vigorous aerobic PA per week and maintain a variety of PA on 3 or more days per week. However, a report showed that approximately 28% of Americans aged 50 and above do not engage in any form of PA, apart from work and daily life, in the past month [2]. The lack of regular PA in older adults [3], which has various negative effects on their overall health and functioning [4, 5]. The absence of PA in older adults leads to a decrease in physical function, mobility [6] and independence [7], resulting in a decrease in quality of life [2] and an increase in healthcare expenses [8]. Therefore, it is important to explore more appealing and effective strategies to encourage older adults to stay physically active.

Intergenerational physical activities have emerged as a promising strategy for addressing the lack of PA among older adults [9]. These projects involve structured and unstructured physical activities [10] that bring together people of different ages and promote interaction, mutual learning, and social support [11, 12]. Intergenerational PA not only provides exercise opportunities for older adults [13], but also promotes intergenerational connections and reduces age-related stereotypes [14]. Intergenerational relationships have been identified as a source of motivation for older adults to engage in regular exercise [15]. Studies [16] have shown that intergenerational PA enhances older adults’ self-esteem, making them more likely to engage in PA. Wu's research has found that verbal or nonverbal interactions between older adults and younger students in intergenerational PA contribute to meaningful intergenerational relationships that enhance social connectedness and reduce social isolation [17].

Although the potential benefits of intergenerational PA, the needs and interests [18–21] of older adults who participate in such activities may be overlooked [18] and participation rates among older adults remain relatively low [19]. To effectively promote and implement such programs, it is crucial to understand the barriers that hinder adults' participation and the facilitators that encourage them to do so.

As far as we know, there is no systematic review of facilitators and barriers to intergenerational PA, and there is a particular lack of perspectives from older adults. Previous systematic reviews have identified barriers and facilitators to PA participation among older adults [20–23], and given the intergenerational context of the current review, where older adults participate in PA alongside children or young people, it is certain the barriers and facilitators to this intergenerational activity are different from other types of PA. Therefore, the purpose of this study is to identify the barriers, facilitating factors, and motivating factors associated with the participation of older adults in intergenerational PA. We will categorize these factors within the theoretical framework of the social–ecological model [24], which includes individual, interpersonal, community, and policy levels [25]. The model is often used to investigate potential factors related to PA in older adults and classify them [26–28]. This model not only takes into account the significance of psychological and social factors in engaging in PA and using PA programs but also considers the role played by organizational, environmental, and policy factors.

## Methods

This review was registered with the International Prospective Register of Systematic Reviews (PROSPERO) and followed the Preferred Reporting Items for Systematic Reviews and Meta-Analysis (PRISMA) guideline [29].

### Inclusion and exclusion criteria

The inclusion criteria were: (a) all types of empirical research designs (quantitative, qualitative, or mixed-methods research) to achieve multiple research objectives; (b) recruiting participants aged 50 and above; (c) PA is defined as any bodily movement produced by skeletal muscles that results in energy expenditure [30]. In this review, intergenerational PA involves promoting health through physical activities and intergenerational learning, such as dancing, yoga, tai chi, etc., with older adults and children or youth together. (d) The main focus is on the barriers and facilitators for older adults to participate in intergenerational physical activities. Barriers refer to individual perceptual barriers to participating in intergenerational public activities, while facilitators refer to characteristics within individuals or their surroundings that motivate intergenerational public activities. (e) Complete papers published in English or Chinese.

The exclusion criteria included: (a) studies without original participant data on barriers and facilitators; (b) studies that are commentaries, editorials, communications, or conference abstracts; (c) elderly individuals with dementia (Alzheimer's disease or related disorders).

### Search strategy

**Fig. 1:**
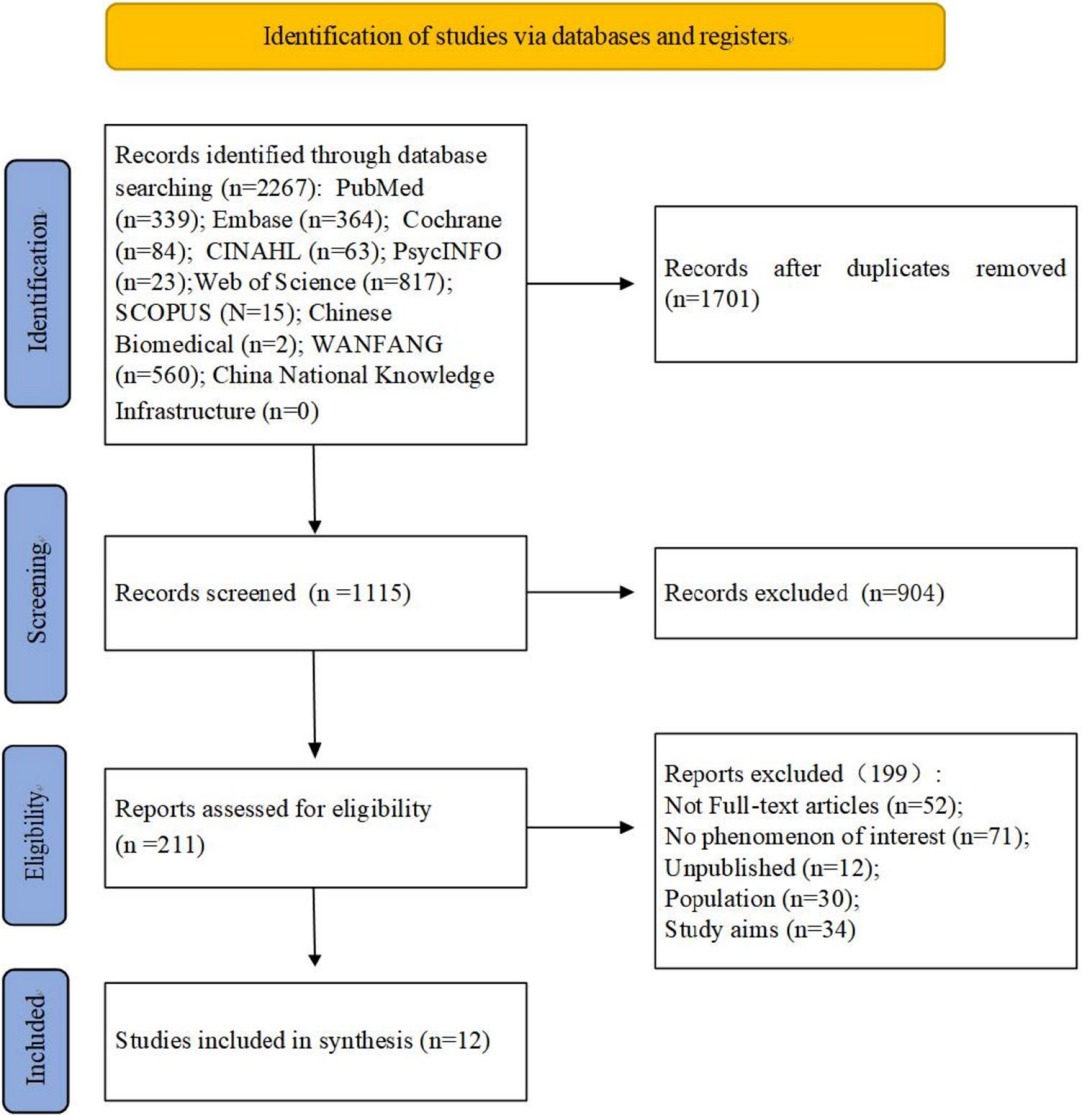
PRISMA flowchart for literature search

The database used: PubMed, Embase, Cochrane Library, Web of Science, SCOPUS, Cumulative Index to Nursing and Allied Health Literature (CINAHL), PsycINFO, China National Knowledge Infrastructure, WANFANG, and Chinese Biomedical. The retrieval time starts from January 2000 to March 2023. The search strategies used are presented in Appendix A. In addition, the reference lists of targeted articles were manually screened for inclusion of eligible studies (Fig. 1).

### Quality appraisal

The Mixed Methods Appraisal Tool (MMAT) is used by two independent assessors to rigorously evaluate the quality of included studies [31]. The MMAT is applicable for assessing various types of research designs, including qualitative, quantitative, and mixed-methods studies. This assessment involves five specific questions for each study design to evaluate the quality of the included research. These questions address the purpose and design of the retrospective study, recruitment strategies, data collection, and analysis methods, presentation and discussion of research findings, and conclusions. To assess the quality of the studies included, we calculated the overall quality score, which is the lowest score of the components of the study. If the overall score of the study is > 4, it is considered "high quality", 3–4 is "moderate quality", and < 3 is "low quality". The final score is determined through discussion and negotiation with the third reviewer (WHY); see Appendix B.

### Data extraction and synthesis

Two researchers (ZF & LLF) extracted data from selected full-text studies. Data were extracted on authors, country of publication, year of publication, participant characteristics (number, age), study site, methods, and primary outcomes, reported barriers, and facilitators. The most relevant and appropriate approach to data synthesis for this review was descriptive narrative synthesis. Because papers with both qualitative and quantitative designs were included, a meta-analysis of the data was deemed inappropriate. Discussions between the two writers (ZF & LLF) cleared any uncertainties or doubtful points. Another author (ZH) cross-checked 20% of the randomly selected data extraction records.

Two researchers (ZF & LLF) carried out the data synthesis. The data were extracted using the social–ecological model adaptation framework [24], which included four levels: individual, interpersonal, community, and policy, and the extracted facilitators and/or barriers to intergenerational PA were organized into descriptive major themes and sub-themes using an inductive approach. The (sub)themes were then classified based on the levels of the social–ecological model. Based on the included studies, the total intergenerational PA intervention was interpreted as a factor that might enhance older adults' continued participation in intergenerational PA due to the inability to distinguish which intervention factors were facilitators and/or hindrances.

The social–ecological model [24] is divided into four levels: (1) individual: characteristics such as personal attitudes, motivation, and self-efficacy; (2) interpersonal: family, friends, peers, and other social support systems, as well as ties to social and cultural practices; (3) community: the weather, environmental, and transportation factors that provide intergenerational PA programs, infrastructure, and resources; and (4) policy: government policies and community-based programs.

## Results

### Study selection

The results of the search, screening, and inclusion of studies are reported using the PRISMA flowchart, as shown in Fig. 1. The database search resulted in 2816 records. After removing duplicates, 1115 studies were screened according to title and abstract relevance, of which 904 did not meet the inclusion criteria. Then, the remaining 211 studies were considered potentially eligible and read in full, and another 199 were excluded for not meeting the inclusion criteria. Finally, 12 studies were included in the review, including one qualitative study, six quantitative studies, and five mixed-methods studies [32–43].

### Study characteristics

Table 1 shows the main characteristics of all 12 papers, all of which were published in English between 2006 and 2020 conducted in 6 countries/regions, the United States (*n* = 7), and one study each in Switzerland, Italy, Spain, Iran, and Canada. These studies provided information on the obstacles and facilitators to older adult groups' participation in intergenerational PA. Most studies used quantitative study methods (*n* = 5) [33–36, 38] and mixed-methods analysis (*n* = 5) [32, 39–42]. One study used a qualitative study method [37].

**Table 1:** Characteristics of the studies included

References	Country	Study design	Study population (*N*)	Setting	Intervention content		Results
					Length	Key activities	
Ramos et al. [32]	USA	Mixed-methods study	Older adults (*N* = 118, Age: 50–79)	N/A	N/A	N/A	(1) Promoting general well-being and health; (2) Enhance the chances for interaction and connections; (3) Foster motivation and energy; (4) Preferences for programming
Minghetti et al. [33]	Switzerland	Quantitative studies non-randomized	Older adults (*N* = 47 age: 81.7); children (*N* = 68, age: 4.9);	N/A	45 min/week for 25-weeks	Dynamic balance exercises and object control skills	Seniors exhibited significant gains in all major physical performance (0.61 < *d* ≤ 2.53) and psychological aspects (0.89 < *d* ≤ 1.20)
Buonsenso et al. [34]	Italy	Survey	participants (*N* = 140, aged 67.8 ± 9.1)	N/A	N/A	PA programs involving both older adults and children	(1) 44.3% show practiced pleasant; (2) 81.5% enjoyed the intergenerational program; (3) PACES-INT for gender (*p* = 0.009), residential location (*p* < 0.001) and employment (*p* = 0.004); (4) 80% would participate in intergenerational programs
Canedo-García et al. [35]	Spain	Survey	participants (*N* = 2013, age: 10–85)	N/A	N/A	Face-to-face activities: healthcare activities;	Physical and mental health, emotions, relationships, self-determination, social involvement, and academic education all improve
Zhong et al. [36]	USA	Cross-sectional study	Older adults (*N* = 455, age: 73.0)	Community	N/A	N/A	Older adults associated with adults and other seniors regularly (79.2%, 1 + times/week), but not as often with children and teenagers;(2) Recreation walking was preferred among older persons (73.3%, 1 + times/week) than transportation walking; (3) Intergenerational activities were significantly predicted by neighborhood perceptions, transportation infrastructure, land uses, land covers, population densities, development activities, and summary scores
Atkins et al. [37]	USA	Qualitative study	participants (*N* = 14; age:18–75)	Community	8 weeks per year, the program over five years	Dance for Health (DFH)	(1) Improving individual well-being; (2) Promoting interpersonal relationships and connections; (3) Fostering community connections
Ebrahimi et al. [38]	Iran	Non-randomized study	Older adults (*N* = 175, age: 71.89 ± 8.3); youth (*N* = 60, age: 21.45 ± 0.3)	Community	Three months, 3 weeks sessions for 90 min/week	Yoga exercise	(1) The intergenerational program and yoga exercises significantly enhanced scores for mental wellness and all of its sub-scales; (2) The intergenerational program had the same effect as yoga exercises with a significant reduction in scores in two sub-scales of social dysfunction and depression
Young et al. [39]	USA	Mix methods study	Grandparents (*N* = 12, age: 62.25); grandchildren (*N* = 23, age: 11.1)	Community	1 h, 2 days/week for 8 weeks	Zumba	(1) The grandparents were more active in the lessons than the grandchildren; (2) Health emerged as a barrier to involvement, but the intergenerational nature of the intervention served as a facilitator; (3) The setting field described how the grandparents' complicated lives affected their ability to participate, and the recreation staff's commitment assisted the intervention to move along smoothly
McConnell et al. [40]	Canada	Mix methods study	Older adults (*N* = 9, age: 72.35); students (*N* = 22, age: 9.86)	School	6-week	Joint “marathon” walking/tracking activity; Chair aerobic; Health fair	(1) Both groups had high levels of satisfaction, leadership confidence, and intention to use what they learned; (2) There was significantly more equipment and organized activities on the playground; (3) Both age groups displayed positive affect during interactions
Strand et al. [41]	USA	Mix methods study	Older adults (*n* = 46, Age: ≥ 60); University students (*n* = 18, age: 19–26)	Rural	24-week, 60 min total weekly	Aerobic, strength train; Fundamental sports; Activities by using Wii EA Active	Effective in promoting PA, socialization, and improving the overall physical health of sedentary older adults in rural areas
Perry and Weatherby [42]	USA	Non-randomized study	Older adults (*N* = 10, age: 60–85); youth (*N* = 8, age: 8–14)	Community	Eight-week, 60 min/week	Tai chi program	More intergenerational connection and greater physical exercise
Ransdell et al. [43]	USA	Randomized controlled trials	Grandmothers (*N* = 11, age:50–70); daughters (*N* = 13, Age: 8–13)	Home	6 months, at least 3 times per week	Home-based PA	Positive improvements in the predicted direction were observed in the home-based group (+ 305% and + 37%, respectively)

### Quality assessment

The MMAT appraisal results are presented in Appendix B. The included studies had methodological quality scores ranging from zero to a maximum score of 5. All studies had a quality score of more than 3, and 3 of the 12 studies scored 5 [33, 36, 38]. The remaining studies scored 4 (*n* = 8) [32, 34, 35, 37, 39, 40, 42, 43] and 3 (*n* = 1) [41]. Generally, the articles that were included exhibited a level of quality ranging from moderate to high.

### Barriers and facilitators

This study focused on different types of intergenerational PA activities, focusing on the barriers and facilitators to participation in these programs among the older population. A social-ecological model was used to report these barriers and facilitators (see Table 2), summarizing them at 4 levels, summarizing 7 main themes and 14 sub-themes, with 32 individual, 14 interpersonal, 23 community, and 4 policy-level (73 in total) facilitators and 20 individual, 4 interpersonal, 11 community, and 2 policy-level (37 in total) barriers, Further details are presented in Appendix C.

**Table 2:** Themes and sub-themes influencing intergenerational PA in older adults within the social-ecological model

Social–ecological model	Theme	Subtheme	Facilitator		Barrier	
			Quantitative	Qualitative	Quantitative	Qualitative
Individual level	1. Knowledge, awareness, and attitude	1.1 Knowledge, awareness, attitude	6	6	1	2
	2. Personal and health factors	2.1 Personal factors	4	1	4	5
		2.2 Emotional status	3	5	1	2
		2.3 Physical health	5	2	3	2
Interpersonal level	3. Social support	3.1 Informal network	5	7	1	3
		3.2 Connection with cultural practices	1	1	0	0
Community level	4. Objective Physical Environments	4.1 Space and Location	4	2	1	0
		4.2 Weather conditions	1	0	0	1
	5. Resources	5.1 Availability and continual access to PA programs	0	2	0	1
		5.2 Equipment	2	2	2	1
		5.3 Service	0	2	0	1
	6. Safety	6.1 Type of sporting program	2	2	0	2
		6.2 Transportation	3	1	0	2
Policy/institutional	7. Organizational factors	7.1 Management of activities	2	2	0	2

#### Individual level

In terms of older adults’ knowledge, awareness and attitude, positive attitudes, enjoyment or interest, and sense of achievement were common facilitators [32, 34, 35, 38, 40, 42], In addition, the need for activity, learning of skills, and perceived benefits of exercise are also facilitators [37, 40, 41]. In terms of personal and health factors, the groups of older women, married persons, housewives, and farmers show a higher interest in group activities, especially intergenerational physical activity [34, 35]. Older adults, particularly elderly women, housewives, and married individuals, have more leisure time and a more flexible schedule, which makes it easier for them to engage in intergenerational physical activities [35]. Engaging in physically challenging and stimulating intergenerational physical activities with children can bring about a sense of youthfulness, vitality, and happiness [32, 34, 40, 42], as well as enhance intelligence and strength and improve overall health [35, 36, 40, 42], which are facilitators of older adults' participation in intergenerational physical activity. For farmers, participation in intergenerational physical activity aligns with the communal spirit in rural areas [33, 44], making it a more attractive option for them [34]. However, these factors are not unique to the groups of older women, housewives, married persons, and farmers; they may be more pronounced in these groups because of their particular life circumstances and social dynamics. At the individual level, physical health conditions such as age differences, fear of fatigue, and physical limitations are considered barriers [32, 34, 35, 39]. Language barriers, lack of motivation, time, and interest discourage older adults from actively participating in activities [32, 37, 41]. Social roles, obligations, and low income, especially for older women who need to prioritize family care, limit their participation in intergenerational PA [39]. Non-compliance or disturbance in the activity and preference of exercise program are also barriers [39, 40].

#### Interpersonal level

Family members, partners, and friends can do PA together as facilitators for older adults to participate in activities [35, 39, 43]. Developing or improving interpersonal relationships, facilitating communication, and motivating each other during the activity are also facilitators [32, 35, 37, 38, 40, 41]. Religious culture or values, such as familism, promote active participation of older adults in intergenerational exercise activities [32, 36]. Disharmonious family relationships [34] and lack of family support [32] were barriers to interpersonal relationships, as were barriers to communication between older adults and children in physical activities and leadership challenges in older adults [40].

#### Community level

Community-level facilitators include those in the built environment, such as those having enough space for exercise [39, 41], communities having a park, fitness center, or equipment [36, 39, 42]. Good weather [42], convenient and safe transportation facilities [36], as well as familiar and easily accessible site [42] help older adults reach sites for intergenerational physical activities. Program sustainability, consistency, such as continued, increased financial investment in the program [37, 40], coaches' fidelity behavior [39], and consistent play programs for older adults and children [40] are also important contributing factors. Certainly, low-difficulty and fun PA programs such as recreational walking [36], and lack of transportation [32, 37] are community-level barriers, such as in urban settings where it is more difficult for older adults to pick up their grandchildren and then go to other sites for activities [34]. Less knowledge/awareness of intergenerational PA programs [32] and barriers to continued older adult participation in intergenerational PA programs, such as technical difficulties associated with exercise games and exercise itself, and a lack of younger adult trainers [41, 42].

#### Policy/institutional level

The development, expansion, and management of activities are important facilitators of the continued participation of older adults [36, 40]. Also, the use of virtual remote technology has helped older adults continue to participate in activities when they are unable to gather in person during the COVID-19 pandemic [40]. In contrast, organizational issues [40] and the relatively short duration of the program [41] were the barriers.

## Discussion

In this systematic review of 12 studies, the barriers and facilitators to intergenerational PA from the perspective of older adults were complex. This study combines these barriers and facilitators by comprehensively identifying them at four levels of the social–ecological model. In this model, factors related to individuals, interpersonal relationships, communities, and policies have become highly influential in the field of older adults in intergenerational PA. The study reveals that barriers are primarily focused on the personal and community levels, with some barriers also present at the interpersonal level. On the other hand, promoting factors are most commonly observed at the personal level, with relatively equal proportions of promoting factors at the interpersonal and community levels.

At the individual level, interest in exercise, enjoyment, and perceived benefits all have a significant impact on encouraging older adults to participate in intergenerational PA. Jenkin’s [45] study also supports this viewpoint, showing that the benefits older adults gain from participating in community PA increase their participation. The intergenerational nature of PA is intriguing and attracts older adults to participate in activities [33, 38]. In these activities, older adults and young people come together to enjoy the benefits of intergenerational exercise [34], leading to improvements in their physical and mental well-being [33]. This finding is similar to Lakicevic's [46] research, which suggests that novel PA measures can enhance participants' interest and enjoyment, motivating them to maintain PA and ultimately achieve better health outcomes. Bethancourt [47] and Franco's study also mentions that the physiological and psychological benefits of PA serve as promoting factors for engaging in PA.

Other personal-level facilitators were experienced when older people engaged in intergenerational PA, so various PA advantages regarding mental and physical wellness have been noted, such as increased self-confidence, accomplishment, well-being, and improved appearance [48], cognition [49], and strength [50]. Perceiving improvements in psychological well-being through enhanced emotions is correlated with the motivation for elderly individuals to engage in physical activities [51]. This viewpoint is also supported by findings in our study. Additionally, self-efficacy plays a crucial role in this process. Royse's research also suggests that exercise can enhance self-efficacy and increase PA, creating a positive cycle that promotes a positive lifestyle [52]. In Wu's research, encouraging the elderly to participate in choreography in intergenerational dance activities not only improves the self-efficacy and sense of accomplishment of the elderly but also improves their creativity and activity motivation [17].

The research emphasizes that the motivation of older adults is an important factor in promoting PA and intergenerational PA. To attract older adults to participate in intergenerational PA, it is necessary to consider older adults' motivation to participate and its significance, which is consistent with the previous studies [27, 51, 53].

The most commonly reported barriers are related to physical condition, discomfort (age and activity differences), and lack of motivation and time. Physical condition has been recognized as a common barrier for older adults participating in physical activities [54, 55], but the physical health of children in intergenerational PA can also affect older adults' ability to continue participating, for example, when grandchildren also experience injury-related health problems and older adults may have to discontinue their activities [56].

Older adults expect the design of intergenerational PA programs to be designed with their age and physical conditions [56]. To guarantee that intergenerational PA and sports programs are acceptable, program planners must first discover the types of PA that older adults are most comfortable participating in [57]. Barriers to older adults’ participation in intergenerational PA show how strongly age and gender-related interpersonal and cultural factors influence individual behavior, as has been found in previous research [58].

Several studies [59–61] have reported similar findings regarding the discomfort that older adults experience in intergenerational programs. These studies have found that older adults often face difficulties in communicating with younger people and encounter inherent ageism, an inability to fully integrate into activities. In the context of intergenerational PA, older adults often experience age-related discomfort due to concerns about fatigue and being unable to keep up with the younger participants [32, 34]. Differences in activity preferences between genders are observed in older adults. Older men tend to prefer physical activities that are more intense or involve competition and outdoor activities, which may lead to a greater inclination of women toward intergenerational activities, especially indoor sports. This conclusion is consistent with Varma's [62] research. However, Gobbi's [63] and Arazi’s [64] study mentioned that the participation rate of women in physical activities may be lower than that of men due to their fear of falling, which could be influenced by cultural and geographical factors. Women face more restrictions in this regard. Additionally, the lack of sufficient time for older adults to engage in intergenerational physical activities may be related to their family responsibilities and conflicting obligations.

Two studies [23, 65] have provided support for this point. The review found that minority groups, particularly women, need to take on more family responsibilities [32, 41].

At the interpersonal level, the support of family and friends is one of the most common facilitators [21, 66]. It is known that social support promotes PA in older adults, and studies have shown that the majority of older adults who participate in intergenerational programs need support and companionship [67]. In May [68] and Brown's [69] study, it was also recommended that family PA opportunities be provided and that families and friends participate in intergenerational PA together to support and encourage each other to keep them active. Similarly, in Jarrott's [70] study, it was proposed to include mechanisms to promote friendship and socialization, provide meaningful roles, and reduce isolation and loneliness in intergenerational sports programs [67].

Fortunately, establishing a good relationship with children can motivate older adults to continue exercising [32, 42], which aligns with Caroline's [71] research findings, positive intergenerational relationships are mutually beneficial, as children gain experience from older adults, while older adults experience a greater sense of self-worth from children. Additionally, both older adults and children exhibit similarities in physical inclinations, such as balance, strength performance [72], and mutual needs in social learning [73].

Furthermore, the participation of elderly individuals in intergenerational PA is closely related to cultural background and customs. Ethnic minorities, particularly elderly women, are more affected by this, while the cultural emphasis on family in Latino communities encourages intergenerational PA participation [32]. A descriptive review [74], also noted the low participation of ethnic minority older women in PA and the need to identify culturally safe community-based PA. Additionally, language is a factor that needs to be considered. Organizing intergenerational sports activities in the participants' primary language can enhance their sense of self-efficacy in participating in these activities.

At the community level, although the promotion factors for elderly participation in intergenerational physical activities outweigh the obstacles, potential barriers such as extreme weather conditions, difficult physical activities, inconvenient transportation, a lack of young coaches, incomplete infrastructure, and technical difficulties still exist. For many older adults, bad weather is a difficult obstacle to overcome and a factor that hinders their ability to participate in outdoor activities [70]. This finding is supported by several studies [75, 76], particularly for residents living in Midwestern cities in the United States [65].

Another barrier factor to participation in intergenerational PA is safety. Unfamiliar locations or a lack of direct transportation often lead to high attrition rates, especially for older adults who rely on walking or public transportation. This finding is supported by the research of Barnett [77] and Lin [78], who systematically examined and quantified the built environment correlates of PA in older adults. Hamer’s [79] study indicates that safety, accessible destinations, and convenient travel paths facilitate older adults' active participation in PA.

Exergaming is an innovative approach that amalgamates PA with video games, and it could offer a fresh experience for older adults, encouraging their interest in intergenerational PA. According to Anderson’s [80] research, older adults appreciated the challenge of playing against virtual trainers as well as the visual stimulus offered by games. This not only helps to focus attention on the enjoyment of electronic games but also gains the health benefits of PA. Phang's [81] study supports this finding by demonstrating that digital intergenerational programs can increase older adults' engagement and reduce loneliness.

However, technological difficulties such as device setup and connectivity pose a challenge for older adults who are unfamiliar with operating such devices. In Chao's study [82], older adults were provided with various levels of exercise games, and this personalized choice helped improve participants' exercise attendance. These issues necessitate consideration and resolution in future intergenerational PA projects' design.

Large scale and long duration are policy-level barriers, which are reflected in other social–ecological levels through high employee turnover. A systematic review [9] supports this finding, indicating that long or large-scale PA increases interference with the existing commitments or daily activities of older adults. Therefore, appropriately reducing the duration of activities may encourage support and interest among older adults. Strand [41] suggests starting with small-scale projects and gradually expanding them to ensure participation and commitment. Therefore, in the future, when formulating intergenerational PA, considering appropriate compression of activity duration may encourage the support and interest of older adults.

### Limitations and prospects

Although this study has made every effort to ensure the rigor of the mixed-methods systematic review and has used the theoretical framework of the social–ecological model to capture barriers and facilitators, there are still some limitations. First, most of the studies included in this systematic review were conducted in high-income countries, thereby omitting crucial factors that may impact middle-income and low-income nations. Furthermore, in our review, the elderly population with cognitive impairments was excluded. However, many of them could benefit from intergenerational programs [11]. Additionally, while a meta-analysis would be ideal, it is not practical owing to the lack of consistency among articles, as well as the disparities in outcomes and samples. Finally, it was observed that, while intergenerational projects are growing in popularity, there is an absence of academic research on specific projects, and most of the research is descriptive and lacks clear results, indicating that further exploration is needed in the field of intergenerational studies.

## Conclusion

In summary, the goal of this research is to better comprehend the barriers and facilitators that older adults face in participating in intergenerational PA from their perspective. Furthermore, this study identifies these barriers and facilitators across multiple levels, which span the interpersonal, policy organizational, and individual levels, providing a crucial reference for future policymakers, researchers, and professionals to create and improve intergenerational PA for this demographic. However, it is worth noting that there is a lack of similar research from middle- and low-income countries, so future studies should consider perspectives from these countries. We suggest that future research should investigate the sustainability and scalability of intergenerational PA and examine specific impacts on different subgroups of older adults, thus having a positive impact on promoting physical, mental, and cognitive health, preventing falls, and modeling behaviors in older adults.

## Data Availability

All the data used and analyzed during the current study are available from the corresponding author upon reasonable request.

## Appendix A: Search strategy and results

### Number of citations by each database register searched

**Table:**

Databases	Citations
Web of Science	817
Embase	364
PubMed	339
CINAHL	63
Cochrane	84
SCOPUS	15
Chinese Biomedical	2
China National Knowledge Infrastructure	0
PsycINFO	23
WANFANG	560
Total (databases)	2267

### Full search strategy for each database

#### PubMed

((intergeneration*[Title/Abstract] OR cross-generation*[Title/Abstract] OR civic engagement[Title/Abstract] OR intergenerational programs[Title/Abstract]) AND (older adult[Title/Abstract] OR elder*[Title/Abstract] OR senior[Title/Abstract] OR aged[Title/Abstract] OR ag?ing[Title/Abstract] OR older person[Title/Abstract] OR older people[Title/Abstract])) AND (activ*[Title/Abstract] OR exercis*[Title/Abstract] OR physical activity[Title/Abstract] OR physical behavior[Title/Abstract] OR fitness[Title/Abstract]).

#### EMBASE

(intergeneration*:ab,ti OR 'cross generation*':ab,ti OR 'civic engagement':ab,ti OR 'intergenerational programs':ab,ti) AND ('older adult':ab,ti OR elder*:ab,ti OR senior:ab,ti OR ag?ing:ab,ti OR aged:ab,ti OR 'older person':ab,ti OR 'older people':ab,ti) AND (activ*:ab,ti OR exercis*:ab,ti OR 'physical activity':ab,ti OR 'physical behavior':ab,ti OR fitness:ab,ti).

#### Cochrane

Intergeneration* OR cross-generation* OR civic engagement OR intergenerational programs in Title Abstract Keyword AND older adult OR elder* OR senior OR aged OR ag?ing OR aged OR older person OR older people in Title Abstract Keyword AND activ* OR exercis* OR physical activity OR physical behavior OR fitness in Title Abstract Keyword—(Word variations have been searched).

#### Web of science

Intergeneration* OR cross-generation* OR civic engagement OR intergenerational programs (Topic) and older adult OR elder* OR senior OR aged OR ag?ing OR aged OR older person OR older people (Topic) and activ* OR exercis* OR physical activity OR physical behavior OR fitness (Topic).

#### SCOPUS

(TITLE-ABS-KEY ( intergeneration* OR cross-generation* OR civic AND engagement OR intergenerational AND programs) AND TITLE-ABS-KEY ( older AND adult OR elder* OR senior OR aged OR ag?ing OR aged OR older AND person OR older AND people) AND TITLE-ABS-KEY ( activ* OR exercis* OR physical AND activity OR physical AND behavior OR fitness)).

#### CINAHL

AB (intergeneration* OR cross-generation* OR civic engagement OR intergenerational programs) AND AB (older adult OR elder* OR senior OR aged OR ag?ing OR aged OR older person OR older people) AND AB (activ* OR exercis* OR physical activity OR physical behavior OR fitness).

#### PsycInfo

Abstract: intergeneration* OR Abstract: cross-generation* OR Abstract: civic engagement OR Abstract: intergenerational programs AND Abstract: older adult OR Abstract: elder* OR Abstract: senior OR Abstract: aged OR Abstract: ag?ing OR Abstract: aged OR Abstract: older person OR Abstract: older people AND Abstract: activ* OR Abstract: exercis* OR Abstract: physical activity OR Abstract: physical behavior OR Abstract: fitness.

### China national knowledge infrastructure

(主题 = 代际 OR 跨代 OR 代际项目 OR 代际融合) AND (主题 = 老年 OR 老年人) AND (主题 = 运动 OR 锻炼 OR 活动).

### WANFANG

主题:(代际 OR 代际支持 OR 跨代 OR 代际融合) and 主题:(老年 OR 老年人) and 主题:(运动 OR 锻炼 OR 活动).

### Chinese biomedical

("代际"[摘要:智能] OR "代际支持"[摘要:智能] OR "跨代"[摘要:智能] OR "代际融合"[摘要:智能]) AND( "老年"[标题:智能] OR "老年人"[标题:智能]) AND( "运动"[标题:智能] OR "锻炼"[标题:智能] OR "活动"[标题:智能]).

## Appendix B: Study quality appraisal

See Table 3.

**Table 3:** Assessment of methodological quality of included studies (*n* = 12) using mixed-methods appraisal tool, version 2018

First author, year	Qualitative studies					Criteria from the mixed-methods appraisal tool, version 2018															Mixed-methods studies					Overall quality score, 0–5
						Quantitative studies																				
						RCT					Non-randomized					Descriptive										
	1.1	1.2	1.3	1.4	1.5	2.1	2.2	2.3	2.4	2.5	3.1	3.2	3.3	3.4	3.5	4.1	4.2	4.3	4.4	4.5	5.1	5.2	5.3	5.4	5.5	
Ramos et al. [32], 2023	Y	Y	Y	N	Y											Y	Y	?	Y	Y	Y	Y	Y	Y	N	4
Minghetti et al. [33], 2021											Y	Y	Y	Y	Y											5
Buonsenso et al. [34], 2021																Y	Y	Y	?	Y						4
Canedo-García et al. [35], 2021																Y	Y	Y	?	Y						4
Zhong et al. [36], 2020																Y	Y	Y	Y	Y						5
Atkins et al. [37], 2019	Y	N	Y	Y	Y																					4
Ebrahimi et al. [38], 2019											Y	Y	Y	Y	Y											5
Young et al. [39], 2016	Y	Y	Y	?	Y											Y	Y	Y	?	Y	Y	Y	Y	Y	N	4
McConnell et al. [40], 2016	Y	?	Y	Y	Y											Y	Y	Y	N	Y	Y	Y	Y	N	N	4
Strand et al. [41], 2014	Y	Y	Y	N	Y											Y	Y	Y	Y	Y	Y	Y	Y	N	N	3
Ransdell et al. [43], 2014						Y	?	Y	Y	Y																4
Perry and Weatherby [42], 2011	Y	?	Y	Y	Y						Y	Y	Y	Y	Y						Y	Y	Y	N	N	4

The overall quality score reflects the number of criteria satisfied, varying from no criteria met to all criteria met (5). For mixed method studies, there are 15 criteria to rate (instead of 5) and the overall quality score is the lowest score of these study components; Y = yes/good, N = no/not good, ? = can't tell/not sure; — = no quality criteria are met; underlined articles are mixed method studies; RCT = Randomized-Controlled Trials; RQ = research question. 1.1 = Is the qualitative approach appropriate to answer the RQ?; 1.2 = 1.2. Are the qualitative data collection methods adequate to address the RQ?; 1.3 = Are the findings adequately derived from the data?; 1.4 = Is the interpretation of results sufficiently substantiated by data?; 1.5 = Is there coherence between qualitative data sources, collection, analysis, and interpretation?; 2.1 = Is randomization appropriately performed?; 2.2 = Are the groups comparable at baseline?; 2.3 = Are there complete outcome data?; 2.4 = Are outcome assessors blinded to the intervention provided?; 2.5 = Did the participants adhere to the assigned intervention?; 3.1 = Are the participants representative of the target population?; 3.2 = Are measurements appropriate regarding both the outcome and intervention/exposure?; 3.3 = Are there complete outcome data?; 3.4 = Are the confounders accounted for in the design and analysis?; 3.5 = During the study period, is the intervention/exposure administered as intended? 4.1 = Is the sampling strategy relevant to address the RQ?; 4.2 = Is the sample representative of the target population?; 4.3 = Are the measurements appropriate?; 4.4 = Is the risk of nonresponse bias low?; 4.5 = Is the statistical analysis appropriate to answer the research question?; 5.1 = Is there an adequate rationale for using a mixed-methods design to address the RQ?; 5.2 = Are the different components of the study effectively integrated to answer the RQ?; 5.3 = Are the outputs of the integration of qualitative and quantitative components adequately interpreted?; 5.4 = Are divergences and inconsistencies between quantitative and qualitative results adequately addressed?; 5.5 = Do the different components of the study adhere to the quality criteria of each tradition of the methods involved?

## Appendix C: Overview of facilitators and barriers in relation to (sub)themes within the social–ecological model

See Table 4.

**Table 4:** Overview of facilitators and barriers in relation to (sub)themes within the social-ecological model

Facilitator^a^		Barrier^a^	
Quantitative studies	Qualitative studies	Quantitative studies	Qualitative studies
**Patient perspective**			
*Individual level*			
1. Knowledge, awareness, and attitude			
1.1 Knowledge, awareness, and attitude			
*6*	*6*	*1*	*2*
Interested in group activities (Ramos et al. [32])	Positive attitude (Ramos et al. [32])	Participation in other exercise classes (Strand et al. [41])	Personal motivation (Ramos et al. [32])
Enjoyment for intergenerational PA (Buonsenso et al. [34])	Education and skills gained (Atkins et al. [37])		Lack of participation/noncompliance (McConnell et al. [40])
Rural residents more interested in new PA proposals (Buonsenso et al. [34])	Found it really fun (McConnell et al. [40])		
Self-determination (Canedo-García et al. [35])	Learning (McConnell et al. [40])		
Changing attitudes (Ebrahimi et al. [38])	Rewarding experience (McConnell et al. [40])		
Confidence and enjoyment (Perry and Weatherby [42])	Perceived exercise benefits (Strand et al. [41])		
2. Personal and health factors			
2.1 personal factors			
*4*	*1*	*4*	*5*
The predisposition of women for intergenerational programs (*p* = 0.009) (Buonsenso et al. [34])	Leadership abilities (McConnell et al. [40])	Language proficiency (Ramos et al. [32])	Have less income; Work schedule (Ramos et al. [32])
Housewives and pensioners (Buonsenso et al. [34])		More accustomed to weekly PA and the “ex-sport people” (Buonsenso et al. [34])	Uncomfortable with age or activity differences (Ramos et al. [32])
Level of education (Buonsenso et al. [34])		limited time (Strand et al. [41])	Low-income residents especially minority women (Atkins et al. [37])
Female, single, or married people (Canedo-García et al. [35])		Moved away (Strand et al. [41])	Outside obligations (Young et al. [39])
			Schedule and location conflicts (Strand et al. [41])
2.2 Emotional status			
*3*	*5*	*1*	*2*
Improved social skills, psychological well-being and the ability to build and maintain social relationships* (MD = 6.62 [0.72; 12.80]) (Minghetti et al. [33])	Feel more accompanied (Ramos et al. [32])	Never feel good enough (Buonsenso et al. [34])	Distractions (Young et al. [39])
Generalized well-being (Buonsenso et al. [34])	Enthusiasm and energy (McConnell et al. [40])		Waning enthusiasm (McConnell et al. [40])
Mental health (Canedo-García et al. [35])	Subjective well-being (McConnell et al. [40])		
	Intellectual, physical, and psychological enhancement (Atkins et al. [37])		
	Feel a little younger (Perry and Weatherby [42])		
2.3 Physical health			
*5*	*2*	*3*	*2*
Increase physical health and functionality parameters* (MD = 2.56 [1.69; 3.31]) (Minghetti et al. [33])	Health (McConnell et al. [40])	Fear of fatigue (Buonsenso et al. [34])	Personal physical limitations (Ramos et al. [32])
Increasing their independence* (MD = − 0.44, CI = − 1.38; 0.44) (Minghetti et al. [33])	Felt stronger (Perry and Weatherby [42])	Difficulty to keep up with children (Buonsenso et al. [34])	health issue (Young et al. [39])
Physical health (Canedo-García et al. [35])		Physical health (Canedo-García et al. [35])	
Neighborhood walkability* (OR = 1.461, *p* = 0.013) (Zhong et al. [36])			
Improving anxiety, depression (Ebrahimi et al. [38])			
*Interpersonal level*			
3. Social support			
3.1 Informal network			
*5*	*7*	*1*	*3*
Relationships (Canedo-García et al. [35])	Kids are more motivated…we motivate each other more (Ramos et al. [32])	Logistic and family relational problems (Buonsenso et al. [34])	Family commitments (Ramos et al. [32])
Social participation (Canedo-García et al. [35])	Fostering interpersonal relationships and connections (Atkins et al. [37])		Program communication (McConnell et al. [40])
Participants with a Friend or close relative (Canedo-García et al. [35])	Include the whole family in an activity (Young et al. [39])		Leadership challenges (McConnell et al. [40])
Living with a partner and/or other relatives (Canedo-García et al. [35])	Relationship building (McConnell et al. [40])		
Improving relationships with others, and building social relationships (Ebrahimi et al. [38])	Extra communication with children (McConnell et al. [40])		
	Socializing and exercising with others (Strand et al. [41])		
	Acquainted with one another (Perry and Weatherby [42])		
3.2 Connection with cultural practices			
*1*	*1*	*0*	*0*
Presence of religious destinations* (OR = 1.587, *p* = 0.045) (Zhong et al. [36])	Latino cultural values, such as familism, interested in activities that could engage the whole family (Ramos et al. [32])		
*Community level*			
4. Objective physical environments			
4.1 Space and location			
*4*	*2*	*1*	*0*
land uses, land covers (Zhong et al. [36])	Providing the space for the activity (Young et al. [39])	In the urban context, it is more difficult to reach different places to pick up the children and go to the sports facilities (Buonsenso et al. [34])	
Had 1.5 + acres of the office land use in the neighborhood* (OR = 2.216, *p* = 0.021) (Zhong et al. [36])	Adequate space to exercise without getting in the way of other activities (Strand et al. [41])		
Presence of trails in parks* (OR = 1.885, *p* = 0.007) (Zhong et al. [36])			
Community organizations and community gyms (Perry and Weatherby [42])			
4.2 Weather conditions			
*1*	*0*	*0*	*1*
Weather improves (Ransdell et al. [43])			Extreme weather (Ramos et al. [32])
5. Resources			
5.1 Availability and continual access to PA programs			
*0*	*2*	*0*	*1*
	The continuing social context of the program (Atkins et al. [37])		Less Knowledge/awareness of programs (Ramos et al. [32])
	One consistent playground game (McConnell et al. [40])		
5.2 Equipment			
*2*	*2*	*2*	*1*
Neighborhood aesthetics* (OR = 1.401, *p* = 0.023) (Zhong et al. [36])	Equipment necessary for the health assessment (Young et al. [39])	Infrastructure (Zhong et al. [36])	Technical difficulties associated with the exergame (Week 8) and exercise itself (Strand et al. [41])
Living in a neighborhood with sports and fitness destinations* (*p* = 0.023) (Zhong et al. [36])	The site had no other opportunities or facilities available for physical activity (Strand et al. [41])	Presence of greenbelts* (OR = 0.580, *p* = 0.018) (Zhong et al. [36])	
5.3 Service			
*0*	*2*	*0*	*1*
	The instructor’s fidelity actions and behaviors (Young et al. [39])		The absence of younger adult trainers (Strand et al. [41])
	Increasing input (McConnell et al. [40])		
6. Safety			
6.1 Type of sporting program			
*2*	*2*	*0*	*2*
Gaming activity (Buonsenso et al. [34])	High level of interest included the Carnival Circle Dance featuring the use of instruments, and the handclapping dance requiring partner interaction (Young et al. [39])		Changes to activities (McConnell et al. [40])
Recreational walking (Zhong et al. [36])	Ballroom, line, or square dancing (Perry and Weatherby [42])		Tai chi needed to concentrate and maintain their balance (Perry and Weatherby [42])
6.2 Transportation			
*3*	*1*	*0*	*2*
Traffic safety* (OR = 0.676, *p* = 0.009) (Zhong et al. [36])	Familiar and easily accessible locations (Strand et al. [41])		Don’t have transportation (Ramos et al. [32])
Street length* (OR = 1.181, *p* = 0.001) (Zhong et al. [36])			Transportation issues (Atkins et al. [37])
The density of transit stops* (OR = 2.592, *p* = 0.013) (Zhong et al. [36])			
*Policy/institutional*			
7. Organizational factors			
7.1 Management of activities			
*2*	*2*	*0*	*2*
The use of virtual and remote technologies facilitated in a time when many people were afraid and/or unable to gather in person (Ramos et al. [32])	Condensing activities into fewer months (McConnell et al. [40])		Organizational problems (McConnell et al. [40])
Development activities (Zhong et al. [36])	Activity expansion and management (McConnell et al. [40])		Program’s short duration (Strand et al. [41])

*PA* physical activity, *MD* mean difference, *CI* confidence interval

*Statistically significant change *p* < 0.05

^a^Number of items according to sub-themes
